# Principles Relevant to Health Research among Indigenous Communities

**DOI:** 10.3390/ijerph120505304

**Published:** 2015-05-19

**Authors:** Francis J. O’Donahoo, Kirstin E. Ross

**Affiliations:** Environmental Health, School of the Environment, Flinders University, Adelaide, SA 5001, Australia; E-Mail: fj.odonahoo@bigpond.com

**Keywords:** health research, engagement, community, Indigenous

## Abstract

Research within Indigenous communities has been criticised for lacking community engagement, for being exploitative, and for poorly explaining the processes of research. To address these concerns, and to ensure ‘best practice’, Jamieson*, et al.* (2012) recently published a summary of principles outlined by the NHMRC (2003) in “one short, accessible document”. Here we expand on Jamieson *et al.*’s paper, which while commendable, lacks emphasis on the contribution that communities themselves can make to the research process and how culturally appropriate engagement, can allow this contribution to be assured, specifically with respect to engagement with remote communities. Engagement started before the research proposal is put forward, and continued after the research is completed, has integrity. We emphasise the value of narratives, of understanding cultural and customary behaviours and leadership, the importance of cultural legitimacy, and of the need for time, not just to allow for delays, but to ensure genuine participatory engagement from all members of the community. We also challenge researchers to consider the outcomes of their research, on the basis that increasing clinical evidence does not always result in better outcomes for the community involved.

## 1. Introduction

Jamieson *et al.* [1] recently published in the Medical Journal of Australia: “*Ten principles relevant to health research among indigenous Australian populations*” based on principles outlined by the National Health and Medical Research Council (NHMRC) Ethical Guidelines for Research among Aboriginal and Torres Strait Islander People [2]. Research within Indigenous communities has been criticised for lacking community engagement, for being exploitative and for inadequately explaining the processes of research to participating communities. Jamieson *et al.* [1] seek to address these concerns. We acknowledge the contribution their paper makes to proper understanding of the demands inherent in health research among Aboriginal and Torres Strait Islander people.

However, the paper lacks emphasis on the contribution that Indigenous communities can make to the research process. In our research into the distribution of the nematode *Strongyloides stercoralis* in dog faeces, our work highlighted the need for the inclusion of additional principles for community engagement (Box 1). In collaboration with members from two remote communities in the Northern Territory we offer expansion on that paper. In particular we emphasise three areas for consideration:

(1) Culturally appropriate engagement, with emphasis on narrative, to ensure Indigenous community contribution;
(2) Recognition of the cultural world view and values of both Indigenous communities and researchers;
(3) Appropriate application of the findings of research, recognising that increasing clinical evidence does not always result in better outcomes for the community.

These areas highlight the necessities of understanding the value of narrative, accepting cultural and customary behaviours, recognising community leadership, allowing time to ensure genuine participatory engagement from all members of the community.

## 2. Culturally Appropriate Engagement

### 2.1. Story-Telling: Recognising the Importance of Initial and On-Going Narratives and Story Telling

Without genuine understanding of the research, authentic permission for the research to take place cannot be granted by the community. It is well established that communities have signed up for, and permitted, research to take place which they do not fully understand, primarily as it is the polite thing to do. Permission without understanding is inherently exploitative.

Research can—must be—explained through narratives which convert the research process into a story which is recognisable, acceptable. Story telling will put the research into an Indigenous world view, giving an Indigenous context to both the theory and practice of the research. For research to be incorporated into Indigenous story-telling, a level of trust must be established between researchers and the community. This confidence will take time to develop, and much of the initial discussion will therefore be focussed on establishing this trust. Story telling also invites researchers to comprehend the way the community sees the research and its place in their stories and dreaming. Time, conversation and cooperation with the community will allow this two-way transaction: translation of western ideas into Indigenous narratives, and enabling researchers to see the place the work has in the community world view.

### 2.2. World View: Recognising that Researchers Bring Particular Attitudes, Values and Expectations to the Research

As Palafox, *et al.* [3] note: “Culturally competent cross-cultural research with Indigenous people requires an understanding and application of Indigenous peoples’ paradigms of health knowledge, science and research”. To encourage cultural legitimacy of the research—to ensure it conforms to the accepted frameworks, principles, rules and standards of a particular culture—requires the researchers first to accept that their own frameworks, principles, rules and standards might be different from those of the community [4]. Researchers’ principles and values are not necessarily better [5,6].

The linear approach to research is a western method: develop an hypothesis, obtain funding, approach the community, undertake the research, write up the research for publication and communicate the research outcomes to the community. This approach does not necessarily meet community expectations. As Sherwood [7] notes, the “ways of viewing health for Indigenous and non-Indigenous Australians are divergent” and that the “current research agenda that is dominated by a Western way of knowing research”. Therefore the outcomes that are most important to the researcher (testing hypotheses, developing new approaches to health care delivery) might be of lesser importance to the community. 

## 3. Customary and Cultural Behaviours

### 3.1. Communicating: Recognising the Importance of Identifying Leadership

Determining who speaks for the community is of critical importance [8]. Who are the Traditional Owners (TOs) for the country? It is possible to go to a house, talk to someone, get permission for the research: that person may not be able to speak for the community, or even for that house. It is imperative to know who to speak to, who an outsider is actually allowed to speak to, and who holds the law. The TOs know the hierarchy: who can speak to whom, who holds the law, who has responsibility for what, and how information should be passed through the community. Researchers need to be open to observing these relationships. Gwynn*, et al.* [9] note “large gaps in non-Indigenous researchers’ understandings of culturally appropriate research partnership”. Determining who speaks for the community is of critical importance. Don’t be afraid to ask. If questions are inappropriate the community will inform the questioner.

### 3.2. Communicating: Allowing Community Ownership of the Research

Recognising that transfer of knowledge through the community will take time. Knowledge and understanding need to pass through the community along established cultural networks. This includes identifying and talking directly to the more passive groups, especially the senior women, to ensure they, and the networks they are parts of, are given a voice. Questions about the research will be passed through these networks, and answers subsequently must also pass through these networks. This ‘back and forward’ process of engaging with the community needs to occur many times to allow questions to evolve as community members approach researchers time and time again. Time must be allocated for this to occur. The Guidelines for Ethical Research in Australian Indigenous Studies [10] while not specifically written for health researchers, provides clear guidance about Indigenous ownership of research.

Interpersonal skills need to be highly developed. Researchers should not assume they are the experts. Within the hierarchy of the community they are not. Researchers should ensure that simplification of discussion does not result in condescension. The success of two-way communication can be measured by the level of engagement of the community. Signs of ineffective communication include lack of two-way interchange; lack of gender, age or disability inclusivity; engagement with only the most vocal members.

### 3.3. Communicating: Achieving Participation

Participation is not the same as being objects of research. Participation presupposes inclusivity. Part of the on-going discussion is to ensure all members of the community have knowledge, understanding and acceptance of the pertinent issues. This presupposes a process of meetings, formal and informal consultations, where research activities are clearly laid out, and potential issues teased out. Effective participation may be judged from verbal and visual clues: what is the feedback? Who is bringing feedback? Clues may be monitored in meetings, formal and informal consultations.

## 4. Application of Research Findings

### 4.1. Communicating: Reporting Research Outcomes

Communication should not stop at the end of the research process. Research outcomes must be communicated back to the community. This may be done in many ways, always using community communication networks.

### 4.2. Ensuring Demonstrable Benefits to the Community

Despite a significant amount of research being undertaken with Indigenous communities, many health outcomes remain unchanged [7,11]. Communities have become accustomed to being told what to think and do by health ‘experts’. In addition, individuals must be allowed the dignity of being knowledgeable about their own health.

How might the results of the research affect the community? If, for example, the outcome is a demonstration of poorer health as measured by a particular index, the research may serve to further stigmatise the community. Indigenous communities are heartily sick of being told they are poorer, sicker and less functional than mainstream Australia. Researchers therefore have responsibilities to ensure that the application of research findings will result in tangible outcomes demonstrable benefits to the community. As noted above, before implementation, time must be allowed for understanding, discussion along traditional lines, and acceptance of any proposed action. 

## 5. Conclusions

This paper argues that researchers undertaking work in Indigenous communities ensure that the engagement process be inclusive and overarching. It is inadequate, indeed patronising, to do less—to hold a meeting, talk about the research proposal and forthwith ask individuals for permission to research them. For appropriate engagement to occur a significant amount of time must be allocated. Funding models need to reflect this principle. Funding which does not include allocation of time should be identified and criticised for this limitation. Researchers must also be prepared to alter (or stop) research which is not able to incorporate the outcomes with sensitivity to the Indigenous community.

Engagement started before the research proposal is put forward, and continued after the research is completed, has integrity. It acknowledges the significance of community customary and cultural mores, and the dignity implicit in genuine two-way communication. It acknowledges the importance of sensitively discussing, and perhaps eventually implementing, any outcomes.

Box 1 A project we are undertaking concerns the distribution of the nematode *Strongyloides stercoralis* in dog faeces. *S. stercoralis* is a parasitic nematode with a high incidence in Indigenous Australian communities [12]. Employing cultural leadership involved firstly sitting and talking with the community leaders and Traditional Owners of the communities. These people were already familiar with the nematodes, having been told about them by the community clinic staff. We showed the leaders the nematode using a field microscope, and although they had seen pictures of them, seeing the moving parasites under the microscope astonished them. This enhanced their support for the research. Building their confidence in our research by engaging with them and showing them the worms gave them confidence to ask questions.

Subsequently this knowledge was rapidly transferred to other members of the communities, when these men went home. Community members asked questions. The leaders then came back with community concerns and ideas. The community related the research to their own knowledge, and questions were raised coming from their own experiences, such as the prevalence of the nematodes in cows and kangaroos. These questions could only be answered by expanding the research. The process was valuable for both the community and the researchers. This demonstrates, practically, how culturally appropriate engagement, customary and cultural behaviour and research findings can be employed to facilitate community understanding and discussion.
